# Genetic risk for major depressive disorder and loneliness in sex-specific associations with coronary artery disease

**DOI:** 10.1038/s41380-019-0614-y

**Published:** 2019-12-03

**Authors:** Jessica Dennis, Julia Sealock, Rebecca T. Levinson, Eric Farber-Eger, Jacob Franco, Sarah Fong, Peter Straub, Donald Hucks, Wen-Liang Song, MacRae F. Linton, Pierre Fontanillas, Sarah L. Elson, Douglas Ruderfer, Abdel Abdellaoui, Sandra Sanchez-Roige, Abraham A. Palmer, Dorret I. Boomsma, Nancy J. Cox, Guanhua Chen, Jonathan D. Mosley, Quinn S. Wells, Lea K. Davis

**Affiliations:** 1 Division of Genetic Medicine, Department of Medicine, Vanderbilt University Medical Center, Nashville, TN USA; 2 Vanderbilt Genetics Institute, Vanderbilt University Medical Center, Nashville, TN USA; 3 Division of Cardiovascular Medicine, Departmesnt of Medicine, Vanderbilt University Medical Center, Nashville, TN USA; 4 Vanderbilt Institute for Clinical and Translational Research, Vanderbilt University Medical Center, Nashville, TN USA; 5 Division of General Internal Medicine and Public Health, Department of Medicine, Vanderbilt University Medical Center, Nashville, TN USA; 6 23andMe, Inc., Mountain View, CA USA; 7 Department of Biological Psychology, Vrije Universiteit, Amsterdam, The Netherlands; 8 Department Psychiatry, Amsterdam UMC, University of Amsterdam, Amsterdam, The Netherlands; 9 Department of Psychiatry, University of California San Diego, La Jolla, CA USA; 10 Institute for Genomic Medicine, University of California San Diego, La Jolla, CA USA; 11 Netherlands Twin Register, Department of Biological Psychology, Vrije Universiteit, Amsterdam, The Netherlands; 12 APH (Amsterdam Public Health) Institute, Amsterdam, The Netherlands; 13 Department of Biostatistics and Medical lnformatics, University of Wisconsin-Madison, Madison, WI USA; 14 Departments of Medicine and Biomedical Informatics, Vanderbilt University Medical Center, Nashville, TN USA

**Keywords:** Predictive markers, Depression, Genetics

## Abstract

Major depressive disorder (MDD) and loneliness are phenotypically and genetically correlated with coronary artery disease (CAD), but whether these associations are explained by pleiotropic genetic variants or shared comorbidities is unclear. To tease apart these scenarios, we first assessed the medical morbidity pattern associated with genetic risk factors for MDD and loneliness by conducting a phenome-wide association study in 18,385 European-ancestry individuals in the Vanderbilt University Medical Center biobank, BioVU. Polygenic scores for MDD and loneliness were developed for each person using previously published meta-GWAS summary statistics, and were tested for association with 882 clinical diagnoses ascertained via billing codes in electronic health records. We discovered strong associations with heart disease diagnoses, and next embarked on targeted analyses of CAD in 3893 cases and 4197 controls. We found odds ratios of 1.11 (95% CI, 1.04–1.18; *P* 8.43 × 10^−4^) and 1.13 (95% CI, 1.07–1.20; *P* 4.51 × 10^−6^) per 1-SD increase in the polygenic scores for MDD and loneliness, respectively. Results were similar in patients without psychiatric symptoms, and the increased risk persisted in females even after adjusting for multiple conventional risk factors and a polygenic score for CAD. In a final sensitivity analysis, we statistically adjusted for the genetic correlation between MDD and loneliness and re-computed polygenic scores. The polygenic score unique to loneliness remained associated with CAD (OR 1.09, 95% CI 1.03–1.15; *P* 0.002), while the polygenic score unique to MDD did not (OR 1.00, 95% CI 0.95–1.06; *P* 0.97). Our replication sample was the Atherosclerosis Risk in Communities (ARIC) cohort of 7197 European-ancestry participants (1598 incident CAD cases). In ARIC, polygenic scores for MDD and loneliness were associated with hazard ratios of 1.07 (95% CI, 0.99–1.14; *P* = 0.07) and 1.07 (1.01–1.15; *P* = 0.03), respectively, and we replicated findings from the BioVU sensitivity analyses. We conclude that genetic risk factors for MDD and loneliness act pleiotropically to increase CAD risk in females.

## Introduction

**Fig. 1:**
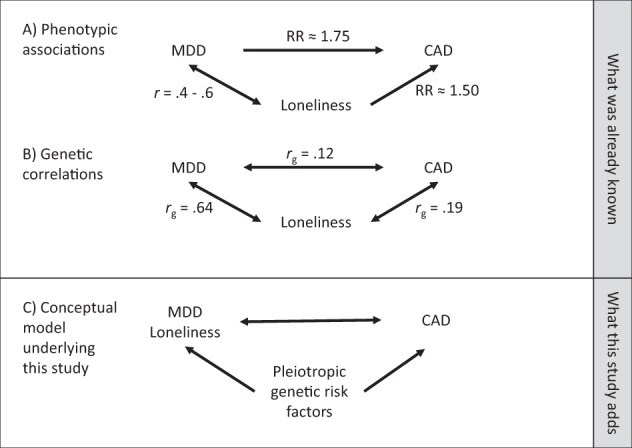
Results from large-scale genome-wide association studies (GWAS) of major depressive disorder (MDD) [11], loneliness [10], and coronary artery disease (CAD) [12] can be used in disentangle relationships between the three traits. **a** Genome-wide genetic correlations show that all three traits share some genetic risk factors. **b** Mendelian randomization experiments using the top associations from GWAS as genetic instruments found no causal effect of CAD on MDD on loneliness, while the reverse tests were likely under-powered. **c** This study used polygenic scores for MDD and loneliness in electronic health records linked to DNA samples and found pleiotropic relationships between MDD, loneliness, and CAD even after accounting for comorbidities and conventional heart disease risk factors

Major depressive disorder (MDD) is among the most common mental disorders, with a lifetime prevalence as high as 17% [1]. MDD and heart disease are closely intertwined. Patients with MDD have an ~75% increased risk of cardiovascular disease [2] and 30–74% of patients who have cardiac events will also meet criteria for MDD (Fig. 1a) [3]. Understanding the complex relationship between these two traits is critical to reducing early mortality, as comorbid MDD is associated with a fivefold increased risk of cardiac mortality within 6 months of a myocardial infarction [3]. In addition to the frequently documented, but poorly understood, relationship between MDD and cardiac health, recent evidence also strongly suggests that chronic loneliness—independent from MDD—similarly increases the risk of heart disease and early mortality. Analyses of ~500,000 participants in the UK biobank showed that loneliness increased the risk of cardiovascular disease incidence by 50% [4] and cardiovascular disease death by 30% [5]. This finding among others [6] has prompted public health initiatives to investigate the physical and mental impact of loneliness. Chronic loneliness reflects a discrepancy between a person’s desired and perceived social connectedness, and is experienced by up to 22% of adults [7].

Loneliness and depressive symptoms are phenotypically correlated (Pearson correlation of 0.4–0.6) [8, 9], but are believed to represent distinct underlying phenomena. Like MDD, loneliness is a heritable trait [10] and the mechanisms linking loneliness to heart disease are incompletely understood.

Summary statistics from large-scale genome-wide association studies (GWAS) show that MDD [11], loneliness [10], and coronary artery disease (CAD) [12], the most common type of heart disease, are genetically correlated, suggesting shared genetic risk factors (Fig. 1b). However, genetic correlations based on GWAS summary statistics can be difficult to interpret, as each study typically ascertains cases on only a single disease without adjusting for or matching on phenotypic comorbidities. While this approach is optimal in GWAS for many reasons, it introduces the possibility of “phenotypic hitchhiking” in which a comorbid trait (e.g., MDD) is unintentionally selected during the ascertainment of the index trait (e.g., CAD). Thus, two heritable phenotypes (e.g., MDD and CAD) that share common environmental risk factors (e.g., obesity) but no genetic risk factors can appear genetically correlated in an analysis based solely on GWAS summary statistics.

Dense, longitudinal clinical data from large genotyped samples provide new opportunities to disentangle shared genetic risk factors from phenotypic hitchhiking. We hypothesized that pleiotropic genetic risk factors in part explained the phenotypic associations and genetic correlations between MDD, loneliness, and CAD (Fig. 1c). We assessed this hypothesis by testing polygenic scores for MDD, loneliness, and CAD for association with clinical diagnoses of MDD and CAD, before and after adjusting for known disease risk factors and comorbidity patterns, in a large collection of patient electronic health records (EHR) linked to genome-wide genotyping. Polygenic scores aggregate the effects of many genetic variants into a single measure of genetic liability to a trait, and can be calculated for all individuals with genome-wide genetic data if their ancestry is similar to the ancestry of the participants in the external GWAS from which the individual genetic variant effect estimates are derived.

This study included five aims. The first was to identify the medical morbidity patterns associated with polygenic scores for MDD and loneliness in the Vanderbilt University Medical Center (VUMC) EHR and accompanying biobank, BioVU. Second, we undertook a targeted analysis of CAD, and determined whether genetic risk for MDD and loneliness remained associated with CAD in the absence of a clinical diagnosis of MDD. Third, we examined whether the risk of CAD conferred by MDD and loneliness risk scores could be attenuated by accounting for multiple conventional CAD risk factors. Fourth, given the well-established sex differences in MDD incidence [1], and in CAD mortality, presentation, and risk factors [13–15], we repeated all analyses in males and females separately. Fifth, we replicated our findings in a large prospectively collected population cohort, the Atherosclerosis Risk in Communities (ARIC) study. Our results provided strong evidence that pleiotropic risk factors for MDD and loneliness increase the risk of CAD, with more pronounced effects in females than in males.

## Methods

### Study populations

The VUMC biobank, BioVU, served as the discovery population. BioVU includes more than 285,000 inpatients and outpatients seen at VUMC whose DNA was extracted from whole blood, and linked to de-identified EHR data spanning 1990–2017 [16]. The BioVU Consent form is provided to patients in the clinic environments at VUMC. The form states policies on data sharing and privacy, and should a signature be obtained, makes any blood leftover from clinical care eligible for BioVU banking. The VUMC Institutional Review Board oversees BioVU and approved this project. The ARIC cohort served as our replication population [17]. The study included 15,792 participants aged 45–64 years in 1987 recruited from four geographic areas in the United States, assessed at baseline for heart disease risk factors, and followed until December 31, 2004 for health outcomes [18]. Use of de-identified ARIC data was approved by the VUMC Institutional Review Board. De-identified ARIC phenotype and genotype data were obtained from dbGaP and analyzed per requirements laid out in the dbGaP Data Use Agreement.

### Genotyping and quality control

A subset of BioVU patients (*n* = 24,262) was genotyped as part of various institutional and investigator-initiated projects on the Illumina MEGA^EX^ platform, which contains more than two million markers. Quality control proceeded as previously described [19]. Genotypes were imputed using SHAPEIT [20]/IMPUTE4 [21] with the 1000 genomes phase I reference panel, and variants with INFO < 0.3 were excluded. A subset of SNPs in linkage disequilibrium was used to calculate relatedness and principal components of ancestry using multidimensional scaling in PLINK v1.9 [22]. We randomly removed one individual from pairs of highly related individuals (pihat > 0.1) to avoid spurious results driven by cryptic relatedness, and restricted to a homogenous population of European descent defined by principal components of ancestry to avoid population stratification effects, leaving 18,385 individuals for analyses. Samples were genotyped in five batches, and variants were removed if allele frequencies differed significantly (*P* < 5 × 10^−5^) between any batch and the rest of the sample. Finally, we filtered multiallelic and structural variants, converted dosage data to hard genotype calls, and excluded variants with certainty <0.9 or INFO < 0.95, resulting in 5,218,407 high quality SNPs across the autosomes.

Genetic data from 13,113 ARIC participants were downloaded from dbGaP (phs000280.v3.p1). Genotypes were measured on the Affymetrix 6.0 SNP array, and data were cleaned as previously described [23]. Genetic ancestry was assigned using STRUCTURE [24] in conjunction with HapMap reference populations, and European ancestry was defined as >90% probability of being in the CEU cluster. Genotypes were imputed to the October 2014 Phase 3 release of the 1000 Genomes cosmopolitan reference haplotypes [25] using SHAPEIT [20]/IMPUTE2 [21] and we excluded SNPs with INFO < 0.7, multiallelic SNPs, and structural variants. Dosage data were converted to hard genotyping calls, excluding variants with certainty <0.9, leaving 6,569,625 high quality SNPs for analysis. Inter-individual relatedness and principal components of ancestry were calculated by GCTA [26] and we randomly excluded one individual from pairs of related individuals (pihat > 0.05), leaving 6975 unrelated European ancestry participants.

### Phenotype data

To facilitate the investigation of comorbidity patterns across the medical phenome, we mapped International Classification of Diseases, ninth edition (ICD-9) billing codes in the EHR to phecodes, which are the higher-order representations of disease categories. ICD-9 codes were mapped to 1814 phecode categories according to the Phecode Map v1.2 (https://phewascatalog.org/phecodes), as implemented in the PheWAS R package v0.12 [27]. Patients were assigned to the case group for a given phecode if they had at least two different ICD-9 codes that mapped to a given phecode, or if they had at least two separate occurrences (i.e., on different days) of a single ICD-9 code that mapped to the given phecode, both of which are validated strategies to improve the positive predictive value of phecodes [28]. The control group excluded patients with only one component ICD-9 code, or with one or more ICD-9 codes that mapped to related phecodes (as defined by the Phecode Map v1.2).

Heart disease and its most common manifestation, CAD, were a focus of our analysis. However, no single phecode was diagnostic of CAD. Therefore, we defined CAD in BioVU patients by a random forest (machine learning) classifier [29] that integrated data from across the EHR (Supplementary Methods; Supplementary Table 1; Supplementary Figs. 1–3). Age in cases was defined by the age of first CAD-defining feature in the EHR, while in controls, age was defined by the age at last encounter. Data on CAD risk factors were also extracted from the EHR either from structured data or via text-mining algorithms (Supplementary Methods). CAD risk factors included body mass index (BMI), hypertension, type 2 diabetes diagnosis, pre-medication blood levels of high- and low-density lipoprotein cholesterol (HDL and LDL) and of triglycerides [30] (lipid-altering medications are listed in Supplementary Table 2), smoking history, and socio-economic status.

A key research question was whether associations with polygenic scores for MDD and loneliness persisted in people without a clinical MDD diagnosis. We defined MDD by the phecode 296.22 “Major depressive disorder”, which included both single episode and recurrent major depressive episodes that are severe, moderate, or of unspecified severity. We also defined broad categories of mental illness, to adjust for the possibility that any degree of mental illness could increase CAD risk simply due to lifestyle factors that may be correlated with having the mental illness itself. We defined a milder depressive symptoms phenotype by one or more depression ICD-9 codes (296.2 and 296.3, including all fifth digit subclassifications, and 311), and as a final test of the robustness of associations, we excluded patients with any psychiatric symptoms (Supplementary Methods). Due to limitations of collecting medication data (e.g., incomplete records, noncompliance, etc.) we did not extract data on psychiatric medications but did model their downstream consequences (i.e., increased BMI).

Phenotype data for ARIC samples came from measurements aggregated by the GENEVA substudy (pht000114.v2.p1), downloaded with permission from dbGaP [23]. Eligible participants were those with data on incident CAD, defined as myocardial infarction, fatal coronary heart disease, silent myocardial infarction detected by electrocardiography, or revascularization procedure. We additionally extracted data on sex, age at first visit, and time to event (CAD or censoring in controls), as well as first visit data on BMI, waist girth, smoking status, hypertension medication use, systolic and diastolic blood pressure, type 2 diabetes diagnosis, highest level of education, use of cholesterol-lowering medication and other medications that secondarily affect cholesterol, and blood measurements of HDL, LDL, and triglycerides (Supplementary Table 3). We defined hypertension by the variable hypertension medication use, or systolic blood pressure >130 mmHg, or diastolic blood pressure >80 mmHg [31]. MDD was not assessed in ARIC participants because ARIC was designed to study cardiovascular outcomes. Therefore, we were only able to test the MDD and loneliness polygenic scores for association with CAD but were not able to adjust those analyses for the presence of clinical MDD.

### Statistical analyses

Polygenic scores for MDD and loneliness were computed using the PRSice software package [32] (Supplementary Methods) for each individual in BioVU and ARIC as follows:

$${\mathrm{Polygenic}}\;{\mathrm{score}} = \mathop {\sum }\limits_{i = 1}^{\# SNPs} (wi\;{\mathrm{x}}\left[ {{\mathrm{SNP}}\;{\mathrm{genotype}}} \right]i)$$

where the SNP genotype was coded as 0, 1 or 2 and w_i_ was the ß coefficient (representing effect size of the marginal association between the SNP and the phenotype) from the Psychiatric Genomics Consortium meta-GWAS of MDD [11], or the Lonely Consortium meta-GWAS of loneliness [10]. We selected SNPs into the polygenic score if their association *P* in the meta-GWAS was below a specified threshold. In the phenome-wide association study, we selected an *P* threshold of 0.05, a value previously found to generate scores that maximized the prediction of MDD [11] and other psychiatric traits [33], and included 392,372 and 379,906 SNPs in the polygenic scores of MDD and loneliness, respectively. In targeted analyses of CAD, we selected the *P* threshold at which the polygenic score was most strongly associated with CAD risk (i.e., at an *P* threshold different than 0.05). This approach improved our power to detect associations with CAD by maximizing the number of pleiotropic SNPs included in the MDD and loneliness polygenic scores. We determined *P* thresholds of 9.0005 × 10^−4^ for MDD (n SNPs = 1387) and 2.50005 × 10^−3^ for loneliness (n SNPs = 2253) by iterating over *P* from 5 × 10^−8^ to 1 in increments of 5 × 10^-5^ and assessing fit via Nagelkerke’s pseudo *R*^2^ (Supplementary Fig. 4). Polygenic scores calculated at these best fit *P* thresholds were used in all sensitivity and replication analyses of CAD to minimize false findings due to overfitting. To adjust for the genetic correlation between MDD and loneliness (Fig. 1b) [10], we used the multitrait-based conditional and joint analysis (mtCOJO) package [34], which uses only meta-GWAS summary statistics to statistically isolate independent genetic associations. Using mtCOJO, we calculated SNP associations with MDD conditional on loneliness (MDD|loneliness), and vice-versa (loneliness|MDD), and calculated polygenic scores from the MDD|loneliness and loneliness|MDD summary statistics using the best fit *P* thresholds above. All polygenic scores were generated by the default pruning and thresholding parameters in PRSice v2 [32] (Supplementary Methods) and were scaled to have an SD of 1. Risk ratios (odds or hazard) reflect the risk increase per 1-SD increase in the polygenic score.

Phenome-wide association studies were conducted with the PheWAS R package v0.12 [27]. We required phecodes to have at least 100 cases, and included covariates for sex, median age across the EHR, genotyping batch, and the first 10 principal components of ancestry.

Targeted analyses of CAD in BioVU employed logistic regression. Minimally adjusted models included covariates for sex, age, genotype batch, and the first ten principal components of ancestry. Fully adjusted models additionally included covariates for BMI, hypertension, smoking, type 2 diabetes, blood measurements of HDL, LDL, and triglycerides, highest level of education, a polygenic score for CAD (Supplementary Methods), and depressive symptoms. In ARIC, we modeled incident CAD using a Cox proportional hazards model and included sex, baseline age, and the first ten principal components of ancestry in the minimally adjusted models. Fully adjusted models included additional covariates for waist girth, smoking status, hypertension medication use, systolic blood pressure, type 2 diabetes, highest level of education, use of cholesterol-lowering medication and other medications that secondarily affect cholesterol, and blood measurements of HDL, LDL, and triglycerides. Although these covariates did not exactly match those we controlled for in BioVU, (i.e., waist girth instead of BMI, hypertension medication use and systolic blood pressure instead of hypertension diagnosis), we used them preferentially since they are better measures of the underlying risk factors, but were rarely reported (waist girth) or error-prone (systolic blood pressure) in the EHR. In the fully adjusted models for both BioVU and ARIC, we tested for interaction between sex and the polygenic score for MDD or loneliness and report interaction *P* < 0.05. In addition, we provide the results of sex-stratified analyses, as effect estimates may differ even in the absence of a statistically significant interaction.

All analyses were completed in R version 3.4.3 or 3.5, and code is available upon request.

## Results

Our analyses included 18,385 BioVU patients. These patients had a median EHR length of 9.91 years (range <1–27 years; Supplementary Table 4), the average median age across the EHR was 57.2 years (SD 17.6), and 50.9% of patients were female. Nearly 5% of patients were diagnosed with MDD, 24.9% had depressive symptoms, and 39.5% had evidence of any psychiatric symptoms. While the genome-wide genetic correlation between MDD and loneliness was 0.64 [10], the polygenic scores for MDD and loneliness had a Pearson correlation of 0.18 (Supplementary Table 5).

**Fig. 2:**
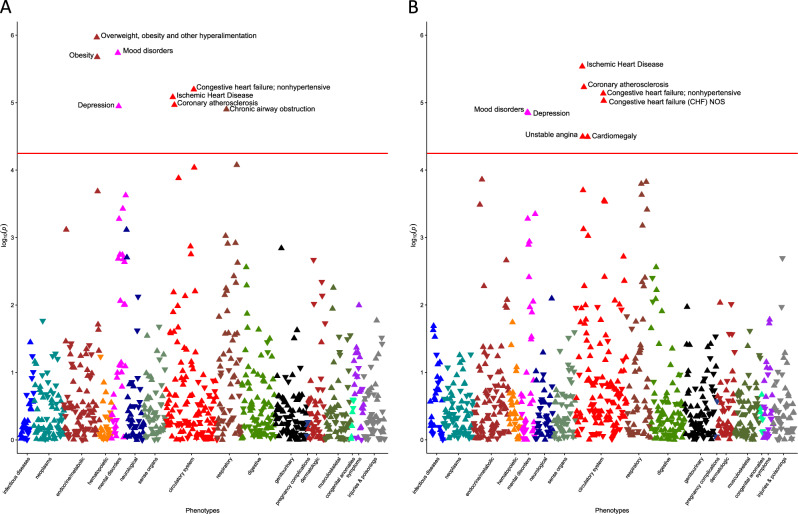
Results from phenome-wide association studies of polygenic scores for MDD (**a**) and loneliness (**b**) in BioVU. The red line denotes the Bonferroni threshold for statistical significance (0.05/882 = 5.67 × 10^−5^), and phenome-wide significant phecodes are labeled. Upward triangles indicate increased odds for a given phecode per 1-SD increased risk in the polygenic score, while downward triangles indicate reduced odds of a given phecode. Interactive plots can be viewed at: https://sealockj.shinyapps.io/mdd_loneliness_cad_interactive/

Our first aim was to identify medical morbidities associated with these polygenic scores using a phenome-wide association study. We analyzed 882 phecodes with at least 100 cases, and used a Bonferroni-corrected phenome-wide significance threshold of 0.05/882 = 5.67 × 10^–5^. This threshold, however, is likely over-conservative because it incorrectly assumes independence between phecodes. The phenome-wide association study of the polygenic score for MDD identified phenome-wide significant associations with “Mood disorders” (odds ratio [OR], 1.10 [95% CI, 1.06-1.15]; *P* = 1.83 × 10^−6^), and “Depression” (OR, 1.10 [95% CI, 1.05–1.15]; *P* = 1.13 × 10^−5^), parent codes for MDD, as expected (Fig. 2a; interactive plots available at: https://sealockj.shinyapps.io/mdd_loneliness_cad_interactive/). The remaining six phecodes that were significant after Bonferroni correction were related to acute and chronic heart diseases and their associated risk factors, including “Ischemic heart disease” (OR, 1.09 [95% CI, 1.05–1.14]; *P* = 8.28 × 10^−^^6^) and “Coronary atherosclerosis” (OR, 1.10 [95% CI, 1.05–1.14]; *P* = 1.08 × 10^−^^5^). The phenome-wide association study of the polygenic score for loneliness (Fig. 2b) also found significant associations with “Mood disorders” (OR, 1.09 [95% CI, 1.05–1.14]; *P* = 1.39 × 10^−5^) and “Depression” (OR, 1.10 [95% CI, 1.05–1.14]; *P* = 1.43 × 10^−5^). The top associations, however, were with heart disease phenotypes, specifically, “Ischemic heart disease” (OR, 1.10 [95% CI, 1.06–1.14]; *P* = 2.91 × 10^−6^) and “Coronary atherosclerosis” (OR, 1.10 [95% CI, 1.06–1.15]; *P* = 5.89 × 10^−6^). In contrast, a polygenic score for CAD (Supplementary Methods) was not associated with MDD (OR, 0.93 [95% CI, 0.87–1.00]; *P* = 0.07), or other mental disorder diagnoses (*P* > 0.05; see above link to interactive plots and Supplementary Fig. 5), replicating previously published findings [35].

**Fig. 3:**
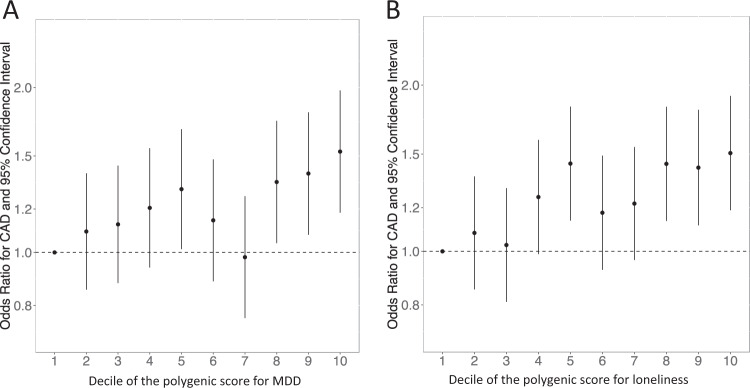
Odds of CAD by decile of the polygenic score for MDD (**a**) and loneliness (**b**) in BioVU. The referent group in all calculations is the lowest polygenic score decile

Motivated by these results, for our second aim, we targeted CAD, to better understand the contribution of genetic risk factors for MDD and loneliness to the shared comorbidity patterns implicating CAD. Our machine learning algorithm identified 3893 cases and 4197 controls in BioVU (Supplementary Table 6). In minimally adjusted models, each SD increase in the polygenic scores for MDD and loneliness, respectively, increased the odds of CAD by 1.11 (95% CI, 1.04–1.18; *P* = 8.43 × 10^−4^) and 1.13 (95% CI, 1.08–1.20; *P* = 4.51 × 10^−6^). These findings mirrored those from the phenome-wide association study and indicated that our results were not biased by the classification ability of our algorithm. Stratified by deciles, patients with polygenic scores for MDD and loneliness in the top versus bottom deciles had, respectively, a 1.53-fold (95% CI, 1.18–1.98; *P* = 1.2 × 10^−3^) and 1.51-fold (95%CI, 1.19–1.91; *P* = 7.4 × 10^−4^) greater risk of CAD (Fig. 3). These associations persisted even after excluding patients with a clinical diagnosis of MDD, depressive symptoms, or any psychiatric symptoms (Table 1).

**Table 1:** Associations between polygenic scores for major depressive disorder (MDD) and loneliness with coronary artery disease (CAD) after excluding patients with a diagnosis of MDD, depressive symptoms, or any psychiatric symptoms

Polygenic score	Patient exclusions	*N* Cases with CAD	N Controls without CAD	OR (95% CI)	*P*
MDD	None	3893	4197	1.11 (1.04–1.18)	8.43 × 10−^4^
MDD	MDD	3744	4037	1.11 (1.05–1.18)	6.54 × 10^−4^
MDD	Depressive symptoms	2935	3147	1.10 (1.03–1.18)	5.76 × 10^−3^
MDD	Any psychiatric symptoms	2440	2551	1.11 (1.03–1.19)	9.27 × 10^−3^
Loneliness	None	3893	4197	1.13 (1.08–1.20)	4.51 × 10^−6^
Loneliness	MDD	3744	4037	1.13 (1.07–1.20)	9.22 × 10^−6^
Loneliness	Depressive symptoms	2935	3147	1.14 (1.08–1.22)	2.08 × 10^−5^
Loneliness	Any psychiatric symptoms	2440	2551	1.12 (1.05–1.20)	8.69 × 10^−4^

*MDD* major depressive disorder

**Fig. 4:**
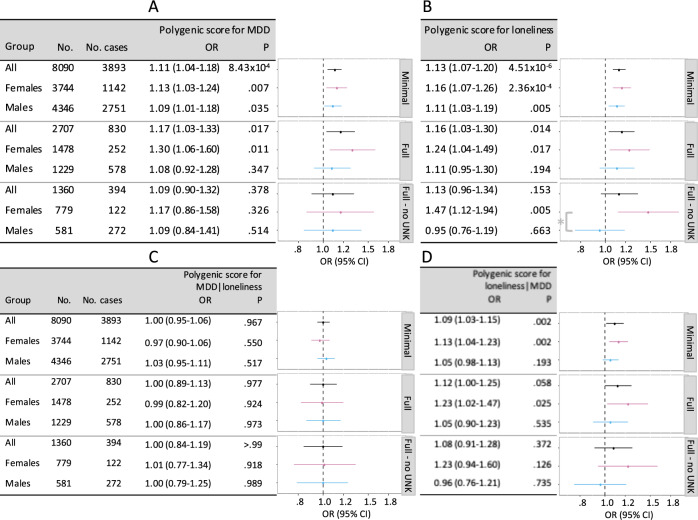
Risk of CAD predicted by polygenic scores for MDD (**a**), loneliness (**b**), MDD|loneliness (**c**), and loneliness|MDD (**d**) before and after adjustment for conventional heart disease risk factors. Minimally adjusted (“Minimal”) models included sex (except sex-stratified results), age, the first ten principal components of ancestry, and genotype batch. Fully adjusted (“Full”) models included additional covariates for BMI, hypertension, smoking, type 2 diabetes, blood measurements of HDL, LDL, and triglycerides, highest level of education, a polygenic score for CAD, and depressive symptoms. Values for smoking and highest level of education were unknown for ~50% of patients. These unknown values were retained in the “Full” model by modeling an explicit category for “missing” values, and were removed from the “Full - no UNK” model. Patients were excluded from the fully adjusted models if there were missing values for any of the other included covariates. The asterisk in **b** denotes sex × polygenic score interaction *P* < .05

For aims 3 and 4, we tested these associations in multivariable models overall, and stratified by sex, and found that associations were robust to adjustment for conventional risk factors in females, but not in males (Fig. 4a, b; polygenic score for loneliness by sex interaction *P* < 0.05). We next accounted for the genetic correlation between MDD and loneliness using the mtCOJO package [34] (Pearson correlations between polygenic scores are in Supplementary Table 5). The polygenic score for MDD|loneliness was not associated with CAD risk (Fig. 4c), whereas the score for loneliness|MDD remained associated with CAD risk in females, even in the fully adjusted model (Fig. 4d). The polygenic score for loneliness|MDD was associated with a 1.23-fold (95% CI, 1.02–1.47; *P* = 0.017) increased odds of CAD in females in the fully adjusted model that maximized patient inclusion by including categories for missing values. The association was equivalent, although less statistically significant (OR, 1.23 [95% CI, 0.94–1.60]; *P* = 0.126) in the fully adjusted model that excluded the 699 female BioVU patients with missing covariate data.

Our fifth aim was to replicate our findings in an independent population. The ARIC sample included 7197 participants of European ancestry with clean genotyping data, followed for a median of 16.0 years (range 0.05–18.1), over which time 923 incident CAD cases were recorded (Supplementary Table 7). The polygenic score for MDD was associated with a hazard ratio (HR) of 1.07 (95% CI, 0.99–1.14; *P* = 0.07), as was the polygenic score for loneliness (HR, 1.07 [95% CI, 1.01–1.15]; *P* = 0.03). Associations were robust to confounder adjustment in females but not in males (Supplementary Fig. 6), and the polygenic score for loneliness|MDD was associated with a CAD HR of 1.14 (95% CI, 1.01–1.29; *P* = 0.02) in females versus 0.98 (95% CI, 1.02–1.29; *P* = 0.60) in males in the fully adjusted model (interaction *P* < 0.05).

## Discussion

This study investigated the phenome-wide consequences of a genetic liability to MDD and loneliness in BioVU, which is a deeply phenotyped large-scale EHR collection with linked genotype data. We discovered strong associations between polygenic scores for MDD and loneliness with heart disease, and our targeted analysis of CAD revealed that patients with polygenic scores in the highest decile for either MDD or loneliness had a more than 50% greater risk of CAD compared with patients with scores in the lowest decile. Conversely, a polygenic score for CAD was not associated with MDD or other psychiatric phenotypes, in accordance with a previous study of the UK biobank population [35]. Epidemiological associations between MDD and loneliness and heart disease are known [36]; here we showed that the association is partly attributable to genetics. Similar results were observed in ARIC participants, who were prospectively surveyed for heart disease outcomes using traditional epidemiological approaches.

An unique feature of our study was the extensive sensitivity analyses that provided more insight into the reported genetic and epidemiological correlations between MDD, loneliness, and CAD. Genetic correlations alone can be difficult to interpret because they may be influenced by phenotypic hitchhiking. For example, if MDD is comorbid with heart disease, then a GWAS of MDD based on prevalent cases will also ascertain patients with heart disease, which could then induce a genetic correlation between MDD and an independent GWAS of heart disease. Any GWAS that ascertains prevalent cases will be similarly enriched for comorbidities. In the absence of large GWAS meta-analyses of incident cases, we tackled this problem by controlling for multiple clinical risk factors, known CAD risk variants, and genetic correlations, resulting in strong evidence that genes conferring risk for MDD and loneliness exert pleiotropic effects on CAD.

MDD and loneliness are genetically correlated [10]. Our conditional analyses, however, suggested that genetic risk factors specific to loneliness increased CAD risk independent of the genetic risk factors that are shared between MDD and loneliness (Fig. 1). This finding is congruent with the physiological effects of chronic loneliness in humans and in animal models [37]. Loneliness induces a state of self-preservation in anticipation of being without the protection of others: it triggers depressive symptoms that signal the need for support and connection from peers [9]; disrupts sleep to maintain a state of alertness at night [38]; raises blood pressure [39]; and activates the hypothalamic pituitary adrenal axis [37, 40–42], which regulates cortisol, a key hormone in stress reactions, metabolism, digestion, immunity, and energy storage. While these responses may be advantageous for being alone in the short term, health problems ensue when loneliness is chronic and the biological response is sustained [37].

Both MDD and heart disease exhibit significant sex differences in prevalence and presentation [1, 13, 15], and heart disease remains the number one killer of females in the United States [14]. Our analyses revealed that genetic risk for MDD and loneliness conferred a higher risk of CAD in females than in males, and that the risk in females was robust to adjustment for the major known risk factors. There are two possible, non-mutually exclusive, explanations for this finding. First, genetic predisposition to MDD or loneliness may be a chronic risk factor for females but an acute risk factor for males, as was previously suggested in a study of 30,000 twins from the Swedish population-based twin registry [43]. Second, the conventional risk factors identified in most epidemiological studies may be poorer predictors of CAD for females than they are for males. Non-traditional and female-specific CAD risk factors (e.g., rheumatoid arthritis, preterm delivery) may contribute to sex differences in CAD prevalence, presentation, and mortality [15], but have only recently been interrogated and so we did not include them in our multivariable models. Future work is warranted to investigate the role of pleiotropic genes on both heart disease and MDD, especially in females where the effects are strongest, and the etiology of heart disease more obscure.

### Limitations

EHR data reflect real clinical use patterns, but some data can be missing or inaccurate. We addressed this potential limitation by implementing a carefully curated CAD phenotype, and conducted sensitivity analyses excluding people with unknown covariate values. In addition, the VUMC inpatient and outpatient population included in our study may be sicker than the general population. Nonetheless, we replicated our findings in a prospective epidemiological cohort ascertained from the general population. Even though MDD was not assessed in ARIC participants, which hampered our ability to replicate results from aim 2, we replicated all other aims and findings, including that polygenic scores for MDD and loneliness were associated with CAD even after adjusting for conventional heart disease risk factors. Another limitation of our study was its restriction to individuals of European ancestry, but this decision was necessitated by the ancestry of patients in the meta-GWAS used to build the polygenic scores. The relevance of our findings to individuals of other ancestries, therefore, is unknown [44, 45].

## Conclusions

Mental health has historically been siloed away from the rest of medicine, resulting in a poor understanding of the relationship between mental and physical health. This EHR-based study showed that genetic risk for MDD and loneliness were just as strongly associated with heart disease as with depression itself. Moreover, the increased risk of CAD persisted in females after adjusting for psychiatric symptoms and multiple other risk factors, suggesting that the excess risk is due to pleiotropic genetic effects and is not simply a behavioral consequence of depression. Identification of these pleiotropic genes could advance both precision medicine and drug development.

## Supplementary information

Supplementary Material

Supplementary Figure 1

Supplementary Figure 2

Supplementary Figure 3

Supplementary Figure 4

Supplementary Figure 5

Supplementary Figure 6
