# Supplementary material for: Genetic risk for major depressive disorder and loneliness in sex-specific associations with coronary artery disease
Source: Mol Psychiatry. 2019 Dec 3;26(8):4254–64. doi: 10.1038/s41380-019-0614-y (PMC7266730; doi:10.1038/s41380-019-0614-y)
Supplement: Supplementary file 1 — Supplementary Material [file 41380_2019_614_MOESM1_ESM.docx]

**Supplementary Online Content**

[**Supplementary Methods** 2](#_Toc5428756)

[**Development of a Machine Learning Classifier for CAD** 2](#_Toc5428757)

[**Extraction of Covariate Data from the EHR** 4](#_Toc5428758)

[**Development of Polygenic Scores** 6](#_Toc5428759)

[**Supplementary Table 1. Features of coronary artery disease used by the random forest classifier to identify cases and controls in BioVU.** 8](#_Toc5428760)

[**Supplementary Table 2. Lipid-lowering medications extracted from the EHR of BioVU patients using MedEX.** 9](#_Toc5428761)

[**Supplementary Table 3. Covariates in the ARIC replication analysis.** 11](#_Toc5428762)

[**Supplementary Table 4. Characteristics of genotyped BioVU patients included in the phenome-wide association study.** 13](#_Toc5428763)

[**Supplementary Table 5. Correlation between polygenic scores.** 14](#_Toc5428764)

[**Supplementary Table 6. Characteristics of BioVU CAD cases and controls.** 15](#_Toc5428765)

[**Supplementary Table 7. Characteristics of ARIC cases and non-cases.** 16](#_Toc5428766)

[**References in Supplementary Material** 17](#_Toc5428767)

# **Supplementary Methods**

## **Development of a Machine Learning Classifier for CAD**

CAD was defined in BioVU subjects by a random forest machine learning classifier^1^ that integrated data from across the EHR: inpatient and outpatient billing codes from the International Classification of Diseases, 9th edition (ICD-9), Current Procedural Terminology (CPT) codes, laboratory values, reports, and clinical documentation. The algorithm was developed in a subset of VUMC patients who were born before 1989 and who had complete demographic information (gender, date of birth, race/ethnicity) listed in their clinical record (Supplementary Figure 1). We also removed patients for whom classification would be trivial, i.e., those whose EHR lacked any CAD features that were used for classification. From this group of 330,802 patients, we randomly selected training (N= 773) and testing (N=294) sets, and the de-identified EHR of these patients was manually reviewed and adjudicated to either a “CAD” or “No CAD” group. The remaining 329,835 patients were assigned to the implementation set.

The random forest method iteratively constructed decision trees using an *a priori* set of defined features to segregate “CAD” and “No CAD” patients in the training set. Feature weights were developed in the training set and were then used to construct a continuous predicted probability (score) of having CAD for each individual in the testing set, where the performance of the classifier was evaluated. The algorithm was built using the Python package ScikitLearn v0.18.1.^2^ Features for algorithm training included 162 CAD-related ICD-9 codes, CPT codes, laboratory values, text strings, and medications. For each feature, 3 metrics were used: counts (the number of times a feature was in a record), persistence (the proportion of record the feature was present), and durability (the proportion of record from the first mention to the end of a record the feature was present). An optimal score threshold (.662) was identified by five-fold cross validation in the testing set, and was selected to yield a false positive rate <1.25%, which prioritized specificity over sensitivity (Supplementary Figure 2). The final model evaluated in the testing set had a positive predictive value of .97, a negative predictive value of .90, a sensitivity of .73, and a specificity of .99. Features that were most strongly predictive of CAD in the final model (features with importances > .001)^1^ are shown in Supplementary Figure 3. The strongest features were related to ICD 414 and its sub-codes (Supplementary Table 1), which capture chronic ischemic heart disease, coronary atherosclerosis, heart transplant/surgery, and aneurysm and dissection of heart. Age at CAD diagnosis was defined in cases by the date at first EHR mention of a CAD-related ICD or CPT code, or a non-negated text mention of coronary artery bypass grafting or stent.

Since the negative predictive value of the algorithm was .90, we applied additional filters to the “No CAD” patients before including them in our control group. First, we only included patients with scores < .20, indicating that < .20 of trees had classified the patient as having CAD. Second, we required “No CAD” patients to have a minimum record length of two years and to be at least 60 years of age at last record so that controls would have had an opportunity to develop CAD. We also augmented the control set with patients who did not require classification by the algorithm (i.e., with no EHR evidence of CAD), and who met the above criteria for record length and age at last record.

We genetically validated our algorithm by comparing the SNP-based heritability (h^2^_SNP_) of CAD in our case-control sample to h^2^_SNP_ estimates that have been previously reported.^3^ We estimated h^2^_SNP_ by restricted maximum likelihood models in the GCTA package v1.9,^4, 5^ and compared our estimate to the h^2^_SNP_ of the CARDIoGRAMplusC4D Consortium dataset, estimated by LD score regression and reported in LD Hub (<http://ldsc.broadinstitute.org/ldhub/>).^3^ The h^2^_SNP_ of CAD in BioVU was .118 (se=.036) on the observed scale, and .107 (se=.033) on the liability scale (computed using a disease prevalence of 6.2%^6^). In comparison, the h^2^_SNP_ of CAD in the CARDIoGRAMplusC4D Consortium dataset was .073 (se=.005) on the observed scale. Although our estimate was slightly higher, the difference was not statistically significant (P=.58).

## **Extraction of Covariate Data from the EHR**

A strength of the EHR is the availability of data collected over the lifespan on risk factors for common complex diseases, and we included variables for many of these risk factors in our analyses. BMI is frequently calculated at an office visit, resulting in multiple measurements for each patient, which we summarized as a single median value. Hypertension was defined by the presence of ICD-9 codes (401*-405*) and ICD-10 codes (I10*-I13*, I16*), and by problem list mentions of “hypertension” or “htn” (excluding “portal hypertension”, “pulmonary hypertension”, “rv hypertension”, “intracranial hypertension”, and “phtn”). Patients with type 2 diabetes were identified using a BioVU algorithm (https://phekb.org/phenotype/type-2-diabetes-demonstration-project )^7^ in which cases had at least one ICD-9 or ICD-10 type 2 diabetes code and mention of non-insulin hypoglycemic medication. Measurements of HDL, LDL, and triglycerides were extracted from laboratory values in the EHR. LDL was calculated by the Friedewald formula.^8^ Observations were filtered if they were outside of 1-200 mg/dL for HDL, 1-400 mg/dL for LDL, and 1-1000 mg/dL for triglycerides, which removed less than 4% of observations for each lipid trait. We also excluded observations in patients <18 years of age and any observations on or after the first mention of lipid-lowering medication (Supplementary Table 2) in the EHR. Medication status was abstracted from free text in clinical notes via an in-house natural language processing tool.^9^ Median lipid values were calculated in patients with multiple observations.

Smoking was defined by data collected on clinic and nursing intake forms, augmented by ICD-9 codes that mapped to the phecode, Tobacco use disorder (318, including all child phecodes; see Phecode Map v1.2: <https://phewascatalog.org/phecodes>). Individuals identified as “never smokers” were required to have been coded as such on their intake forms, *and* could have no ICD-9 codes indicative of tobacco use disorder. Current or previous smokers were defined as those who had any evidence of smoking in their EHR including clinical notes or ICD-9 codes indicative of tobacco use disorder. Patients for whom a determination of “never smoker” or “current or previous smoker” could not be made were assigned an “unknown” smoking status so that they could be included in multivariable models. Socio-economic status was determined by proxy based on the highest level of education abstracted from the EHR by a natural language processing algorithm.^10^ Patients for whom socio-economic status could not be determined by the algorithm were assigned a value of “unknown” so that they could be included in multivariable models.

Psychiatric symptoms were defined by the presence of one or more ICD-9 codes that mapped to phecodes indicative of psychological disorders (295-306.99). Parent phecodes and their descriptions are: Schizophrenia and other psychotic disorders (295), Mood disorders (296), Suicidal ideation or attempt (297), Anxiety disorders (300), Personality disorders (301), Sexual and gender dysphoria (302), Psychogenic and somatoform disorders (303), Adjustment reaction (304), Eating disorder (305.2), and Other mental disorders (306).

## **Development of Polygenic Scores**

We calculated polygenic scores via the pruning and thresholding method implemented in PRSice v2.^11^ SNPs in the meta-GWAS were pruned using an algorithm that scanned over a 250 kb window and removed SNPs in linkage disequilibrium (r^2^>0.1) with the most associated SNP (i.e., lowest P), thus retaining the strongest trait-associated SNP within each linkage disequilibrium block. The algorithm cycled through all SNPs in the meta-GWAS, beginning with the SNP with the lowest P and ending at the SNP whose P exceeded a provided threshold, only allowing each SNP to appear in one clump. Linkage disequilibrium estimates were derived directly from the BioVU genotype data, and only pruned SNPs were used in calculating polygenic scores.

In addition to polygenic scores for MDD and loneliness, we constructed two polygenic scores for CAD, one to be used in a PheWAS, and the other as a covariate in multivariable models. Meta-GWAS summary statistics were obtained from the CARDIoGRAMplusC4D Consortium GWAMA of CAD.^12^ The polygenic score used in the PheWAS was computed at a P threshold of .05, for consistency with the polygenic scores for MDD and loneliness that were tested in PheWAS. The polygenic score included in multivariable models was intended to maximize prediction of CAD in our data, and was computed at a P threshold of 5.0005x10^-4^, selected by iterating over *P* thresholds from 5x10^-8^ to 1 in increments of 5x10^-5^ and assessing fit with CAD in BioVU via Nagelkerke’s pseudo R^2^. The polygenic score calculated at this threshold included 684 SNPs, explained 2.11% of the observed phenotypic variance (2.07% on the liability scale), and was strongly associated with CAD risk (OR, 1.46 [95% CI, 1.38-1.55], P=4.43x10^-41^) per 1-SD increase, in models adjusting for sex, age, batch, and the first 10 principal components of ancestry. Our polygenic score for CAD was calculated at a lower P threshold and included fewer SNPs than recently derived polygenic predictors for CAD.^13, 14^ Many BioVU patients, however, used lipid-lowering medication, which reportedly offsets a high polygenic risk for CAD,^13^ and since the primary purpose of the polygenic score for CAD was to maximize genetic prediction of CAD in our sample, our choice of P threshold was appropriate.

**Supplementary Table 1. Features of coronary artery disease used by the random forest classifier to identify cases and controls in BioVU.** Features included inpatient and outpatient billing codes from the International Classification of Diseases, 9th edition (ICD-9), Current Procedural Terminology (CPT) codes, laboratory values, medications extracted from the EHR using MedEX,9 and keywords extracted from reports and clinical documentation. All ICD-9 codes were grouped to their parent code (i.e., all digits following the decimal were truncated). Each keyword is mapped to its own group, except for the “cath”, “TTE”, and “TEE” groups.

| **Category** | **Features** |
| --- | --- |
| ICD9 Codes | 410, 410.01, 410.02, 410.1, 410.11, 410.12, 410.2, 410.21, 410.22, 410.3, 410.31, 410.32, 410.4, 410.41, 410.42, 410.5, 410.51, 410.52, 410.6, 410.61, 410.62, 410.7, 410.71, 410.72, 410.8, 410.81, 410.82, 410.9, 410.91, 410.92, 410.1, 410.11, 411.81, 411.89, 411.89, 412, 413, 413.1, 413.9, 414, 414.01, 414.02, 414.03, 414.04, 414.05, 414.06, 414.07, 414.1, 414.11, 414.12, 414.19, 414.2, 414.3, 414.4, 414.8, 414.9, 429.79, 996.03, 0.24, 0.66, 17.55, 36.03, 36.03, 36.04, 36.06, 36.07, 36.09, 36.1, 36.11, 36.12, 36.13, 36.14, 36.15, 36.16, 36.17, 36.19, 36.2, 36.31, 36.32, 36.33, 36.34, 36.39, 36.91, 36.99, 37.22, 37.23, 88.5, 88.51, 88.52, 88.53, 88.54, 88.55, 88.56, 88.57 |
| CPT Codes | 33510, 33511, 33512, 33513, 33514, 33515, 33516, 33517, 33518, 33519, 33520, 33521, 33522, 33523, 33534, 33535, 33536, 92920, 92921, 92922, 92923, 92924, 92925, 92926, 92927, 92928, 92929, 92930, 92931, 92932, 92933, 92934, 92935, 92936, 92937, 92938, 92939, 92940, 92941, 92942, 92943, 92944, 92945, 92946, 92947, 92948, 92949, 92950, 92951, 92952, 92953, 92954, 92955, 92956, 92957, 92958, 92959, 92960, 92961, 92962, 92963, 92964, 92965, 92966, 92967, 92968, 92969, 92970, 92971, 92972, 92973, 92974, 92975, 92976, 92977, 92980, 92981, 92982, 92984, 92995, 92996 |
| Laboratory Measures | TropnT, CKMBRe |
| Keywords | stenosis, LHC (group: cath), heart catheterization (group: cath), heart cath (group: cath), LAD stenosis, left anteriod descending artery stenosis, circumflex artery stenosis, LCA stenosis, left coronary artery stenosis, right coronary artery stenosis, RCA stenosis, wall motion abnormality, wall motion abnormalities, blockage, stent, angiogram, stress test, cardiac MRI, TTE (group: tte), transthoracic echocardiogram (group: tte), TEE (group: tee), transesophageal echocardiogram (group: tee), coronary catheterization, coronary cath, ct angiography, tpa, thrombolytics, PCI, percutaneous coronary intervention, Coronary artery bypass grafting, CABG, CAB, Cardiac surgery |
| Medications | aspirin, ASA, dipyridamole, Plavix, clopidogrel, Brilinta, ticagrelor, prasugrel, effient |

**Supplementary Table 2. Lipid-lowering medications extracted from the EHR of BioVU patients using MedEX.**^9^

| **Medication Class** | **Brand Name** | **Generic Name** |
| --- | --- | --- |
| bile acid sequestrants | colestid | colestipol |
| bile acid sequestrants | prevalite | colestyramine |
| bile acid sequestrants | questran | colestyramine |
| bile acid sequestrants | welchol | colesevelam |
| cholesterol absorption inhibitors | zetia | ezetimibe |
| fibric acid derivatives | antara | fenofibrate |
| fibric acid derivatives | antara | fenofibrate |
| fibric acid derivatives | clofibrate | fenofibrate |
| fibric acid derivatives | fenoglide | fenofibrate |
| fibric acid derivatives | fibricor | fenofibrate |
| fibric acid derivatives | lipofen | fenofibrate |
| fibric acid derivatives | lofibra | fenofibrate |
| fibric acid derivatives | lopid | gemfibrozil |
| fibric acid derivatives | triglide | fenofibrate |
| fibric acid derivatives | trilipix | fenofibrate |
| nicotinic acid/niacin | niacor | niacin |
| nicotinic acid/niacin | niaspan | niacin |
| PCSK9 inhibitors | praluent | alirocumab |
| PCSK9 inhibitors | repatha | evolocumab |
| probucol | probucol | probucol |
| statin | advicor | niacin extended-release/simvastatin |
| statin | altoprev | extended-release/lovastatin |
| statin | baycol | cerivastatin |
| statin | caduet | amlodipine/atorvastatin |
| statin | canef | fluvastatin |
| statin | crestor | rosuvastatin |
| statin | juvisync | sitagliptin/simvastatin |
| statin | lescol | fluvastatin |
| statin | lescol xl | fluvastatin |
| statin | lipitor | atorvastatin |
| statin | lipobay | cerivastatin |
| statin | liptruzet | ezetimibe/atorvastatin |
| statin | livalo | pitavastatin |
| statin | mevacor | lovastatin |
| statin | pravachol | pravastatin |
| statin | selektine | pravastatin |
| statin | simcor | niacin extended-release/simvastatin |
| statin | statins | statins |
| statin | vastin | fluvastatin |
| statin | vytorin | ezetimibe/simvastatin |
| statin | zocor | simvastatin |

**Supplementary Table 3. Covariates in the ARIC replication analysis.** A description of variables can be found at: <ftp://ftp.ncbi.nlm.nih.gov/dbgap/studies/phs000280/phs000280.v3.p1/pheno_variable_summaries/phs000280.v3.pht000114.v2.GENEVA_ARIC_Subject_Phenotypes.data_dict.xml>.

| **Variable** | **Description** | **Units** | **Coded values** |
| --- | --- | --- | --- |
| anta07a | Waist girth to nearest cm at visit 1 | cm |  |
| bmi01 | Body mass index in kg/(m*m) at visit 1 | kg/(m*m) |  |
| cholmdcode01 | Cholesterol-lowering medication within 2 weeks |  | 1=Yes 0=No |
| cholmdcode02 | Medications which secondarily affect cholesterol |  | 1=Yes 0=No |
| cigt01 | Cigarette smoking status at visit 1 |  | 1=Current smoker  2=Former smoker  3=Never smoker  4=Unknown, but one of the other 3 categories may be ruled out |
| diabts03 | Diabetes with fasting glucose cut point 126mg/dL at visit 1 |  | 1=Fasting glucose >=126 mg/dL or non-fasting glucose >=200mg/dL or self-report of physician diagnosis or took diabetic medication in previous two weeks 0=All negative |
| elevel01 | Education level, definition 1, at visit 1 |  | 1=Grade school or 0 years education 2=High school, but no degree 3=High school graduate 4=Vocational school 5=College 6=Graduate school or Professional school |
| hdlsiu02 | Re-calibrated HDL cholesterol in mmol/L | mmol/L |  |
| hyptmdcode01 | Hypertension lowering medication use; definition 1, at visit 1 |  | 1=Yes 0=No |
| ldlsiu02 | Re-calibrated LDL cholesterol in mmol/L | mmol/L |  |
| sbpa21 | Systolic blood pressure (average of 2nd and 3rd readings) at visit 1 | mm Hg |  |
| sbpa22 | Diastolic blood pressure (average of 2nd and 3rd readings) at visit 1 | mm Hg |  |
| trgsiu01 | Total triglycerides in mmol/L | mmol/L |  |

# **Supplementary Table 4. Characteristics of genotyped BioVU patients included in the phenome-wide association study.**

|  | BioVU (N=18,385) |
| --- | --- |
| Length of medical record in years, median (min, max) | 9.91 (0, 27.06) |
| Median age across medical record, mean (sd) | 57.20 (17.58) |
| Female, No. (%) | 9355 (50.9) |
| MDD diagnosis, No. (%) | 826 (4.5) |
| Depressive symptoms, No. (%) | 4576 (24.9) |
| Any psychiatric symptoms, No. (%) | 7267 (39.5) |

MDD denotes major depressive disorder.

# **Supplementary Table 5. Correlation between polygenic scores.**

| **Polygenic score 1** | **Polygenic score 2** | **Pearson correlation** |
| --- | --- | --- |
| MDD | Loneliness | 0.177 |
| MDD | MDD\|loneliness | 0.547 |
| MDD | Loneliness\|MDD | -0.001 |
| MDD | CAD | 0.020 |
| Loneliness | MDD\|loneliness | 0.004 |
| Loneliness | Loneliness\|MDD | 0.658 |
| Loneliness | CAD | 0.011 |
| MDD\|loneliness | Loneliness\|MDD | -0.153 |
| MDD\|loneliness | CAD | 0.035 |
| Loneliness\|MDD | CAD | 0.004 |

# **Supplementary Table 6. Characteristics of BioVU CAD cases and controls.**

|  | CAD cases (N=3893) | CAD controls (N=4197) | *P*^a^ |
| --- | --- | --- | --- |
| Age, mean (SD) | 63.4 (11.0) | 71.1 (8.0) | <2.20E-16 |
| Female, No. (%) | 1142 (29.3) | 2602 (62.0) | <2.20E-16 |
| MDD diagnosis, No. (%) | 149 (3.8) | 160 (3.8) | 1.96E-01 |
| Depression diagnosis, No. (%) | 958 (24.6) | 1050 (25.0) | 7.49E-01 |
| Other psychiatric disorder diagnosis, No. (%) | 495 (12.7) | 596 (14.2) | 2.77E-02 |
| BMI, mean (SD) | 29.31 (5.73) | 28.71 (6.10) | 2.78E-05 |
| BMI group |  |  | 1.48E-02 |
| Normal (BMI<25), No. (%) | 828 (21.3) | 1158 (27.6) |  |
| Overweight (BMI≥25 and BMI<30), No. (%) | 1332 (34.2) | 1397 (33.3) |  |
| Obese (BMI≥30), No. (%) | 1441 (34.2) | 1399 (33.3) |  |
| Unknown, No. (%) | 292 (7.5) | 243 (5.8) |  |
| Smoking status |  |  | <2.20E-16 |
| Never, No. (%) | 1084 (27.8) | 1945 (46.3) |  |
| Current or former, No. (%) | 1644 (42.2) | 1075 (25.6) |  |
| Unknown, No. (%) | 1165 (29.9) | 1177 (28.0) |  |
| Hypertension diagnosis, No. (%) | 3715 (95.4) | 3133 (74.6) | <2.20E-16 |
| Type 2 diabetes diagnosis, No. (%) | 426 (10.9) | 238 (5.7) | <2.20E-16 |
| Highest level of education |  |  | 5.50E-12 |
| Less than high school, No. (%) | 159 (4.1) | 96 (2.3) |  |
| High school, No. (%) | 925 (23.8) | 1108 (26.4) |  |
| Bachelor's degree, No. (%) | 194 (5.0) | 260 (6.2) |  |
| Graduate school, No. (%) | 481 (12.4) | 584 (13.9) |  |
| Unknown, No. (%) | 2134 (54.8) | 2149 (51.2) |  |
| Antilipemic medication use, No. (%) | 3773 (96.9) | 2479 (59.1) | <2.20E-16 |
| Pre-medication median blood HDL in mg/dL, mean (SD) | 42.7 (14.78) | 56.55 (19.10) | <2.20E-16 |
| Pre-medication median blood LDL in mg/dL, mean (SD) | 117.1 (37.86) | 119.2 (33.08) | 6.87E-01 |
| Pre-medication median triglycerides in mg/dL, mean (SD) | 191.9 (117.23) | 148.1 (99.25) | 7.16E-15 |

^a^*P* from associations in a logistic regression model adjusted for age and sex.

**Supplementary Table 7. Characteristics of ARIC cases and non-cases.** Values were reported at first visit (i.e., “visit 1”), unless otherwise noted.

|  | **Incident CAD cases (N=923)** | **Non-cases (N=6274)** | ***P*^a^** |
| --- | --- | --- | --- |
| Age at baseline, mean (sd) | 55.6 (5.4) | 53.9 (5.7) | <2.20E-16 |
| Age at event, mean (sd) | 65.1 (6.7) | 69.5 (5.9) | NA |
| Female, N (%) | 287 (31.1) | 3656 (58.3) | <2.20E-16 |
| BMI, mean (sd) | 27.9 (4.70) | 26.7 (4.7) | 1.10E-10 |
| BMI group |  |  | 2.91E-09 |
| Normal (BMI<25), N (%) | 239 (25.9) | 2535 (40.4) |  |
| Overweight (BMI≥25 and BMI<30), N (%) | 259 (28.1) | 2460 (39.2) |  |
| Obese (BMI≥30), N (%) | 423 (45.8) | 1275 (20.3) |  |
| Unknown | 2 (0.2) | 4 (0.1) |  |
| Waist girth in cm, mean (sd) | 100.0 (12.4) | 94.8 (13.2) | 2.26E-13 |
| Smoking status |  |  | 2.43E-15 |
| Never, N (%) | 254 (27.5) | 2654 (42.3) |  |
| Former, N (%) | 378 (41.0) | 2140 (34.1) |  |
| Current, N (%) | 291 (31.5) | 2654 (42.3) |  |
| Unknown, N (%) | 0 | 4 (0.1) |  |
| Hypertension diagnosis^b^, N (%) | 507 (54.9) | 2366 (37.7) | <2.20E-16 |
| Systolic blood pressure, mean (sd) | 124.4 (17.9) | 117.2 (16.3) | <2.20E-16 |
| Diastolic blood pressure, mean (sd) | 73.7 (11.0) | 71.4 (9.7) | 1.87E-04 |
| Type 2 diabetes diagnosis, N (%) | 179 (19.4) | 380 (6.1) | <2.20E-16 |
| Highest Level of Education |  |  | 4.57E-08 |
| Less than high school | 57 (6.2) | 219 (3.5) |  |
| High School | 440 (47.7) | 2777 (44.3) |  |
| Bachelor's Degree | 353 (38.2) | 2626 (41.9) |  |
| Graduate School | 73 (7.9) | 642 (10.2) |  |
| Unknown | 0 | 10 (0.2) |  |
| Cholesterol-lowering medication use within 2 weeks, N (%) | 41 (4.4) | 176 (2.8) | 1.54E-02 |
| Medications that secondarily affect cholesterol | 240 (26.0) | 1142 (18.2) | 2.94E-09 |
| HDL in mg/dL, mean (sd) | 43.5 (12.9) | 52.6 (17.0) | <2.20E-16 |
| LDL in mg/dL, mean (sd) | 148.7 (36.9) | 134.5 (37.3) | <2.20E-16 |
| Triglycerides in mg/dL, mean (sd) | 163.9 (112.7) | 129.6 (87.6) | <2.20E-16 |

^a^*P* from associations in a Cox proportional hazards model adjusted for baseline age and sex.

^b^Hypertension at baseline in ARIC participants was defined according to recent criteria for the detection of high blood pressure^15^ using the criteria: sbpa21>130 or sbpa22>80 or hyptmdcode01=1.

# **References in Supplementary Material**

1. Breiman L. Random Forests. *Machine Learning* 2001; **45**(1)**:** 5-32.

2. Pedregosa FV, G.; Gramfort, A.; Michel, V.; Thirion, B.; Grisel, O.; Blondel, M.; Prettenhofer, P.; Weiss, R.; Dubourg, V.; Vanderplas, J.; Passos, A.; Cournapeau, D.; Brucher, M.; Perrot, M.; Duchesnay, E.;. Scikit-learn: Machine Learning in Python. *Journal of Machine Learning Research* 2011; **12:** 6.

3. Zheng J, Erzurumluoglu AM, Elsworth BL, Kemp JP, Howe L, Haycock PC *et al.* LD Hub: a centralized database and web interface to perform LD score regression that maximizes the potential of summary level GWAS data for SNP heritability and genetic correlation analysis. *Bioinformatics* 2017; **33**(2)**:** 272-279.

4. Yang J, Lee SH, Goddard ME, Visscher PM. GCTA: a tool for genome-wide complex trait analysis. *Am J Hum Genet* 2011; **88**(1)**:** 76-82.

5. Lee SH, Wray NR, Goddard ME, Visscher PM. Estimating missing heritability for disease from genome-wide association studies. *Am J Hum Genet* 2011; **88**(3)**:** 294-305.

6. Writing Group M, Mozaffarian D, Benjamin EJ, Go AS, Arnett DK, Blaha MJ *et al.* Heart Disease and Stroke Statistics-2016 Update: A Report From the American Heart Association. *Circulation* 2016; **133**(4)**:** e38-360.

7. Ritchie MD, Denny JC, Crawford DC, Ramirez AH, Weiner JB, Pulley JM *et al.* Robust replication of genotype-phenotype associations across multiple diseases in an electronic medical record. *Am J Hum Genet* 2010; **86**(4)**:** 560-572.

8. Friedewald WT, Levy RI, Fredrickson DS. Estimation of the concentration of low-density lipoprotein cholesterol in plasma, without use of the preparative ultracentrifuge. *Clin Chem* 1972; **18**(6)**:** 499-502.

9. Xu H, Stenner SP, Doan S, Johnson KB, Waitman LR, Denny JC. MedEx: a medication information extraction system for clinical narratives. *J Am Med Inform Assoc* 2010; **17**(1)**:** 19-24.

10. Hollister BM, Restrepo NA, Farber-Eger E, Crawford DC, Aldrich MC, Non A. Development and Performance of Text-Mining Algorithms to Extract Socioeconomic Status from De-Identified Electronic Health Records. *Pac Symp Biocomput* 2017; **22:** 230-241.

11. Euesden J, Lewis CM, O'Reilly PF. PRSice: Polygenic Risk Score software. *Bioinformatics* 2015; **31**(9)**:** 1466-1468.

12. Nikpay M, Goel A, Won HH, Hall LM, Willenborg C, Kanoni S *et al.* A comprehensive 1,000 Genomes-based genome-wide association meta-analysis of coronary artery disease. *Nat Genet* 2015; **47**(10)**:** 1121-1130.

13. Khera AV, Chaffin M, Aragam KG, Haas ME, Roselli C, Choi SH *et al.* Genome-wide polygenic scores for common diseases identify individuals with risk equivalent to monogenic mutations. *Nat Genet* 2018; **50**(9)**:** 1219-1224.

14. Abraham G, Havulinna AS, Bhalala OG, Byars SG, De Livera AM, Yetukuri L *et al.* Genomic prediction of coronary heart disease. *Eur Heart J* 2016; **37**(43)**:** 3267-3278.

15. Whelton PK, Carey RM, Aronow WS, Casey DE, Jr., Collins KJ, Dennison Himmelfarb C *et al.* 2017 ACC/AHA/AAPA/ABC/ACPM/AGS/APhA/ASH/ASPC/NMA/PCNA Guideline for the Prevention, Detection, Evaluation, and Management of High Blood Pressure in Adults: A Report of the American College of Cardiology/American Heart Association Task Force on Clinical Practice Guidelines. *J Am Coll Cardiol* 2018; **71**(19)**:** e127-e248.
