# Supplementary material for: Genetic risk for major depressive disorder and loneliness in sex-specific associations with coronary artery disease
Source: Mol Psychiatry. 2019 Dec 3;26(8):4254–64. doi: 10.1038/s41380-019-0614-y (PMC7266730; doi:10.1038/s41380-019-0614-y)
Supplement: Supplementary file 2 — Supplementary Figure 1 [file 41380_2019_614_MOESM2_ESM.pptx]

## Slide 1
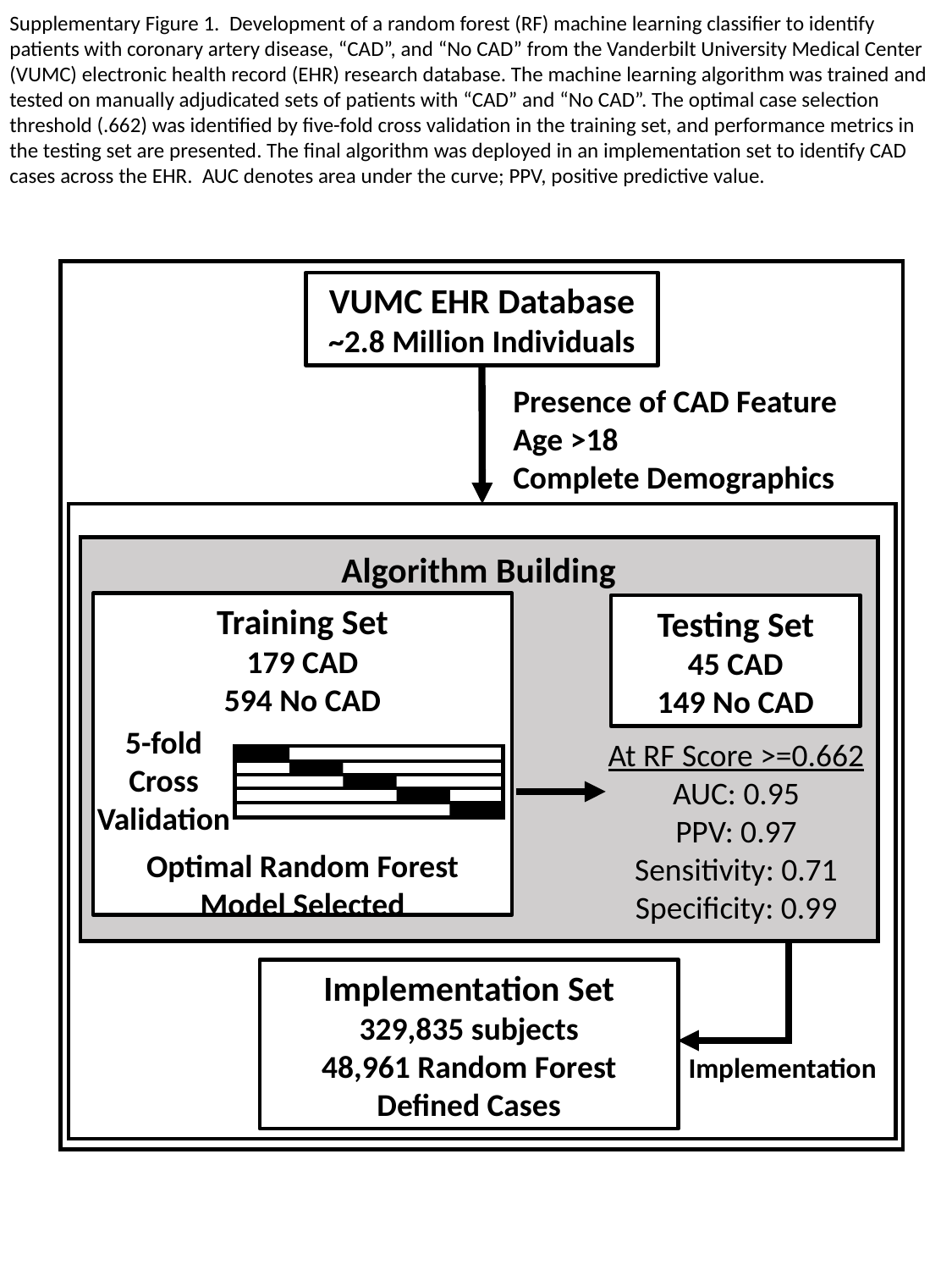

Supplementary Figure 1. Development of a random forest (RF) machine learning classifier to identify patients with coronary artery disease, “CAD”, and “No CAD” from the Vanderbilt University Medical Center (VUMC) electronic health record (EHR) research database. The machine learning algorithm was trained and tested on manually adjudicated sets of patients with “CAD” and “No CAD”. The optimal case selection threshold (.662) was identified by five-fold cross validation in the training set, and performance metrics in the testing set are presented. The final algorithm was deployed in an implementation set to identify CAD cases across the EHR. AUC denotes area under the curve; PPV, positive predictive value.
VUMC EHR Database
~2.8 Million Individuals
Presence of CAD Feature
Age >18
Complete Demographics
Algorithm Building
Training Set
179 CAD
594 No CAD
Testing Set
45 CAD
149 No CAD
5-fold Cross Validation
At RF Score >=0.662
AUC: 0.95
PPV: 0.97
Sensitivity: 0.71
Specificity: 0.99
Optimal Random Forest Model Selected
Implementation Set
329,835 subjects
48,961 Random Forest Defined Cases
Implementation
