# Supplementary material for: Genetic risk for major depressive disorder and loneliness in sex-specific associations with coronary artery disease
Source: Mol Psychiatry. 2019 Dec 3;26(8):4254–64. doi: 10.1038/s41380-019-0614-y (PMC7266730; doi:10.1038/s41380-019-0614-y)
Supplement: Supplementary file 3 — Supplementary Figure 2 [file 41380_2019_614_MOESM3_ESM.pptx]

## Slide 1
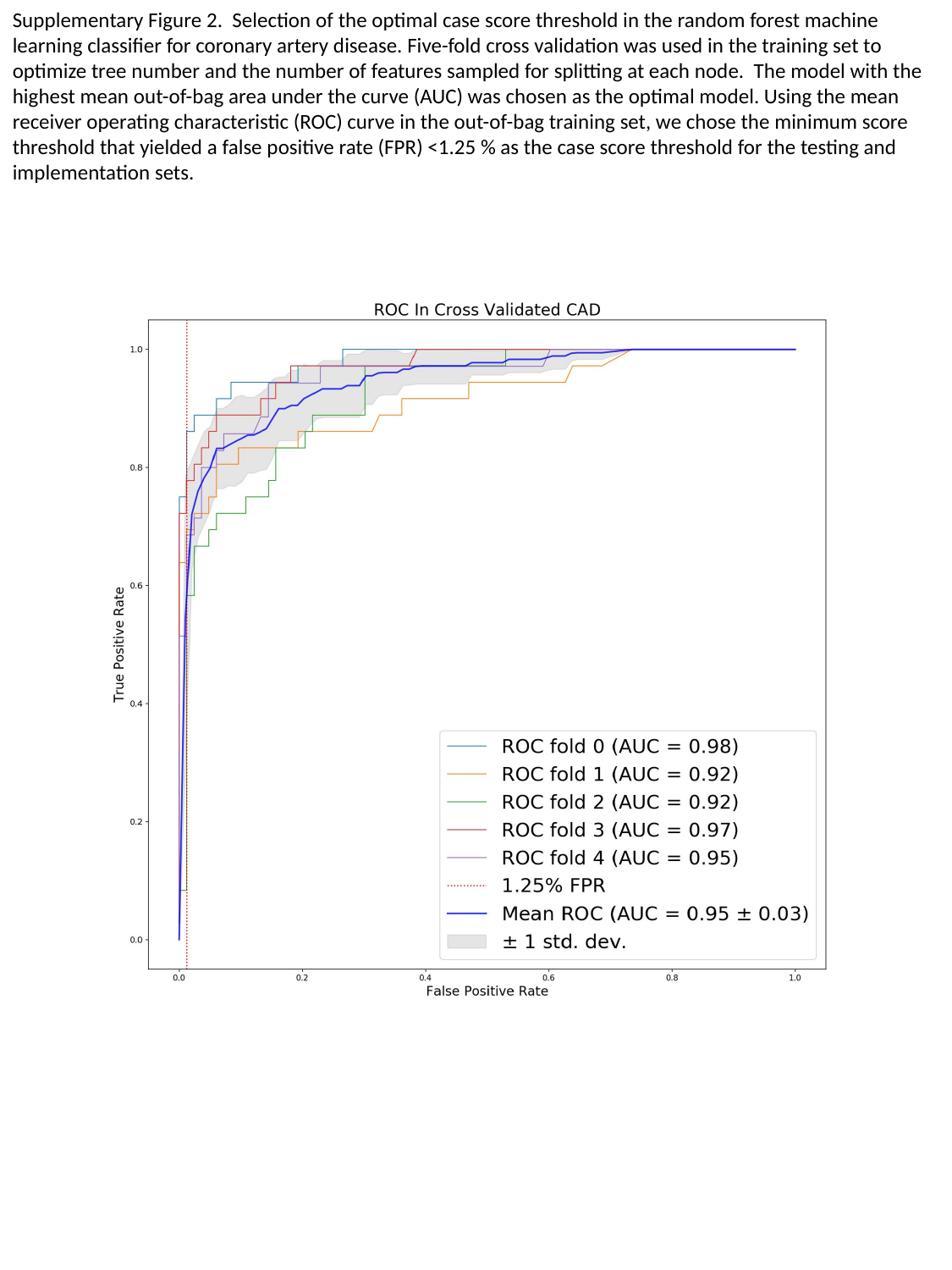

Supplementary Figure 2. Selection of the optimal case score threshold in the random forest machine learning classifier for coronary artery disease. Five-fold cross validation was used in the training set to optimize tree number and the number of features sampled for splitting at each node. The model with the highest mean out-of-bag area under the curve (AUC) was chosen as the optimal model. Using the mean receiver operating characteristic (ROC) curve in the out-of-bag training set, we chose the minimum score threshold that yielded a false positive rate (FPR) <1.25 % as the case score threshold for the testing and implementation sets.
