# Supplementary material for: Genetic risk for major depressive disorder and loneliness in sex-specific associations with coronary artery disease
Source: Mol Psychiatry. 2019 Dec 3;26(8):4254–64. doi: 10.1038/s41380-019-0614-y (PMC7266730; doi:10.1038/s41380-019-0614-y)
Supplement: Supplementary file 4 — Supplementary Figure 3 [file 41380_2019_614_MOESM4_ESM.pptx]

## Slide 1
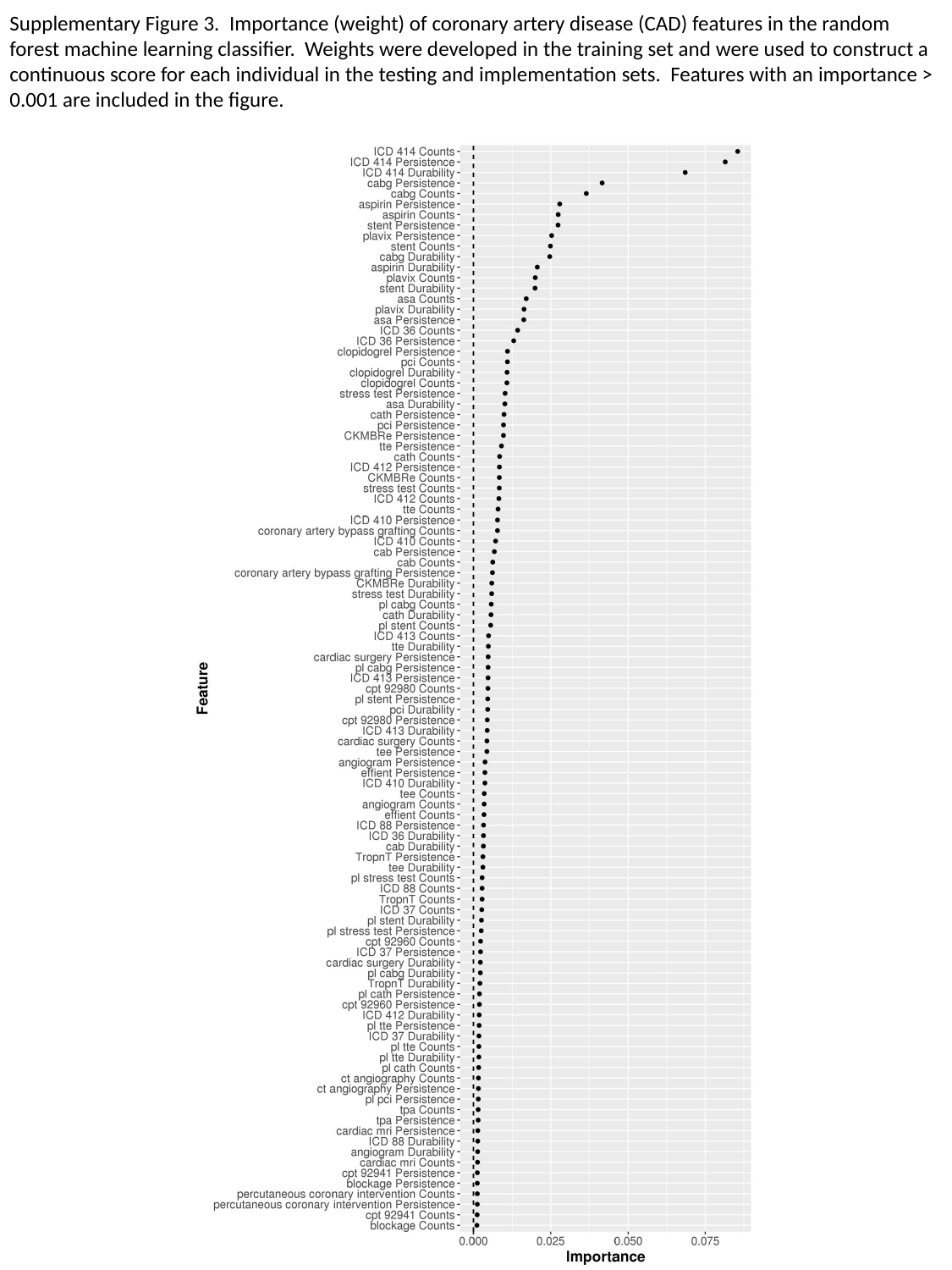

Supplementary Figure 3. Importance (weight) of coronary artery disease (CAD) features in the random forest machine learning classifier. Weights were developed in the training set and were used to construct a continuous score for each individual in the testing and implementation sets. Features with an importance > 0.001 are included in the figure.
