# Supplementary material for: Genetic risk for major depressive disorder and loneliness in sex-specific associations with coronary artery disease
Source: Mol Psychiatry. 2019 Dec 3;26(8):4254–64. doi: 10.1038/s41380-019-0614-y (PMC7266730; doi:10.1038/s41380-019-0614-y)
Supplement: Supplementary file 5 — Supplementary Figure 4 [file 41380_2019_614_MOESM5_ESM.pptx]

## Slide 1
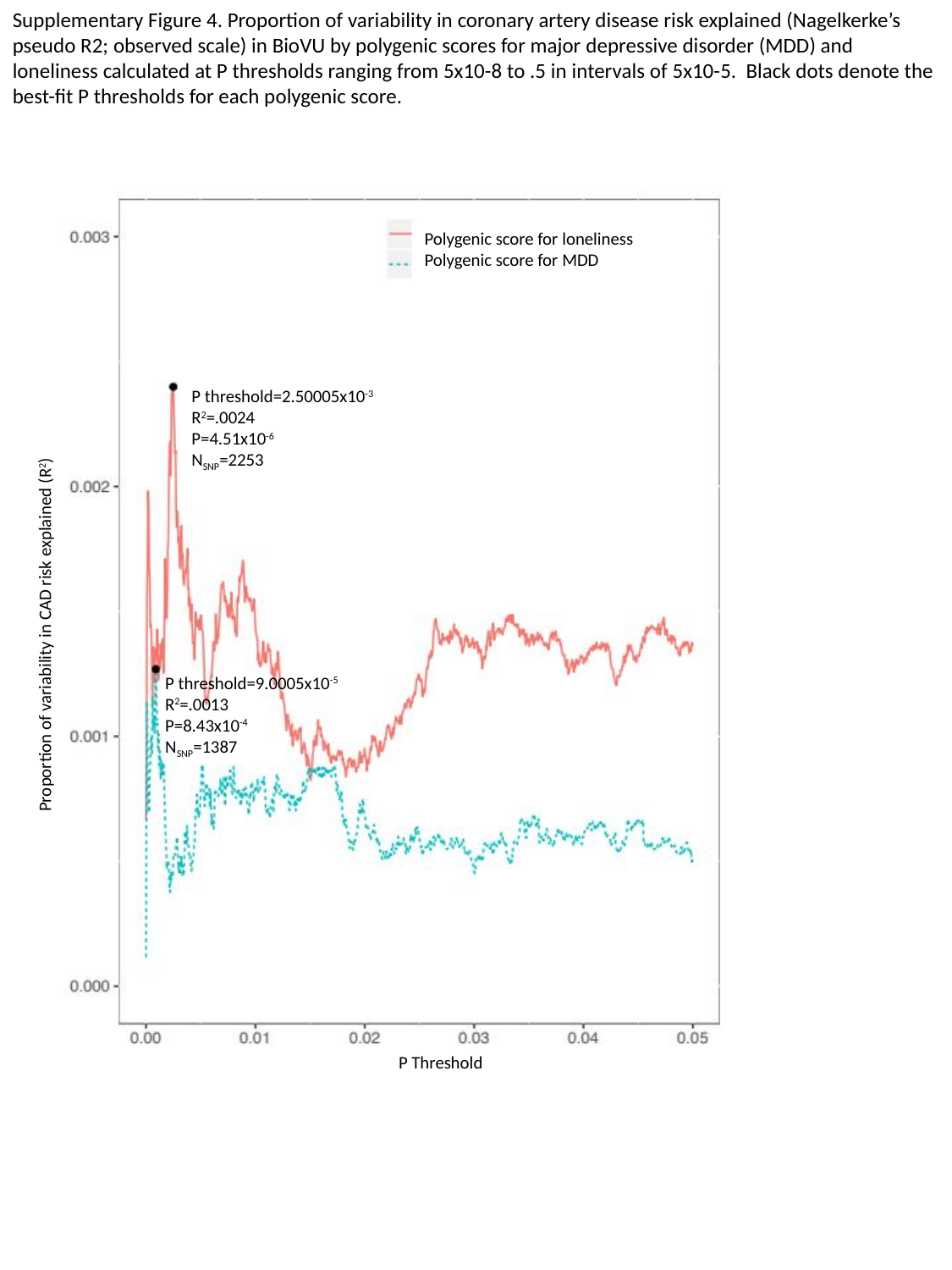

Supplementary Figure 4. Proportion of variability in coronary artery disease risk explained (Nagelkerke’s pseudo R2; observed scale) in BioVU by polygenic scores for major depressive disorder (MDD) and loneliness calculated at P thresholds ranging from 5x10^-8^ to .5 in intervals of 5x10^-5^. Black dots denote the best-fit P thresholds for each polygenic score.
Polygenic score for loneliness
Polygenic score for MDD
Proportion of variability in CAD risk explained (R2)
P threshold=2.50005x10-3
R2=.0024
P=4.51x10^-6^
NSNP=2253
P threshold=9.0005x10^-5^
R2=.0013
P=8.43x10^-4^
NSNP=1387
P Threshold
