# Supplementary material for: Genetic risk for major depressive disorder and loneliness in sex-specific associations with coronary artery disease
Source: Mol Psychiatry. 2019 Dec 3;26(8):4254–64. doi: 10.1038/s41380-019-0614-y (PMC7266730; doi:10.1038/s41380-019-0614-y)
Supplement: Supplementary file 6 — Supplementary Figure 5 [file 41380_2019_614_MOESM6_ESM.pptx]

## Slide 1
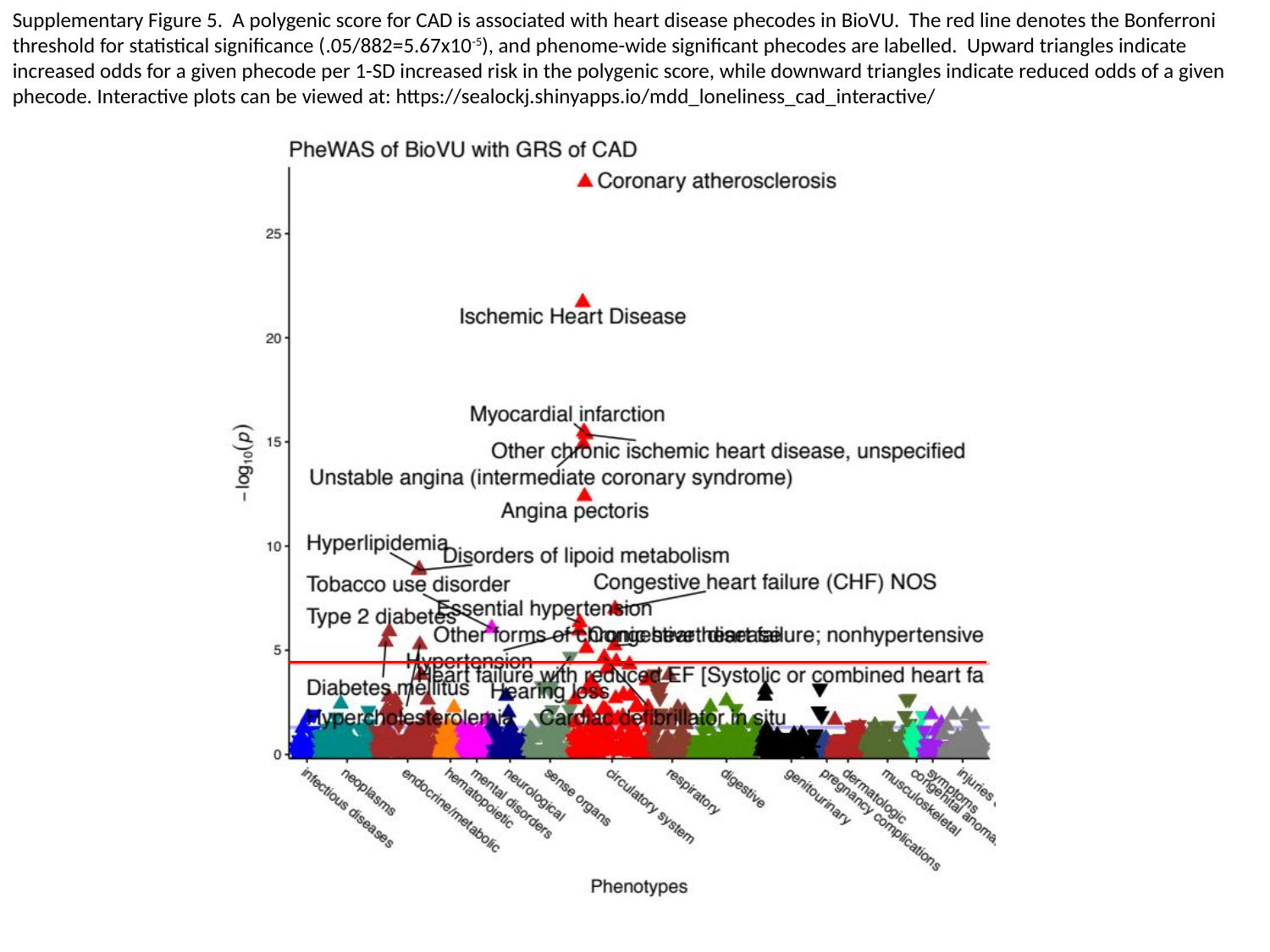

Supplementary Figure 5. A polygenic score for CAD is associated with heart disease phecodes in BioVU. The red line denotes the Bonferroni threshold for statistical significance (.05/882=5.67x10^-5^), and phenome-wide significant phecodes are labelled. Upward triangles indicate increased odds for a given phecode per 1-SD increased risk in the polygenic score, while downward triangles indicate reduced odds of a given phecode. Interactive plots can be viewed at: https://sealockj.shinyapps.io/mdd_loneliness_cad_interactive/
