# Supplementary material for: Genetic risk for major depressive disorder and loneliness in sex-specific associations with coronary artery disease
Source: Mol Psychiatry. 2019 Dec 3;26(8):4254–64. doi: 10.1038/s41380-019-0614-y (PMC7266730; doi:10.1038/s41380-019-0614-y)
Supplement: Supplementary file 7 — Supplementary Figure 6 [file 41380_2019_614_MOESM7_ESM.pptx]

## Slide 1
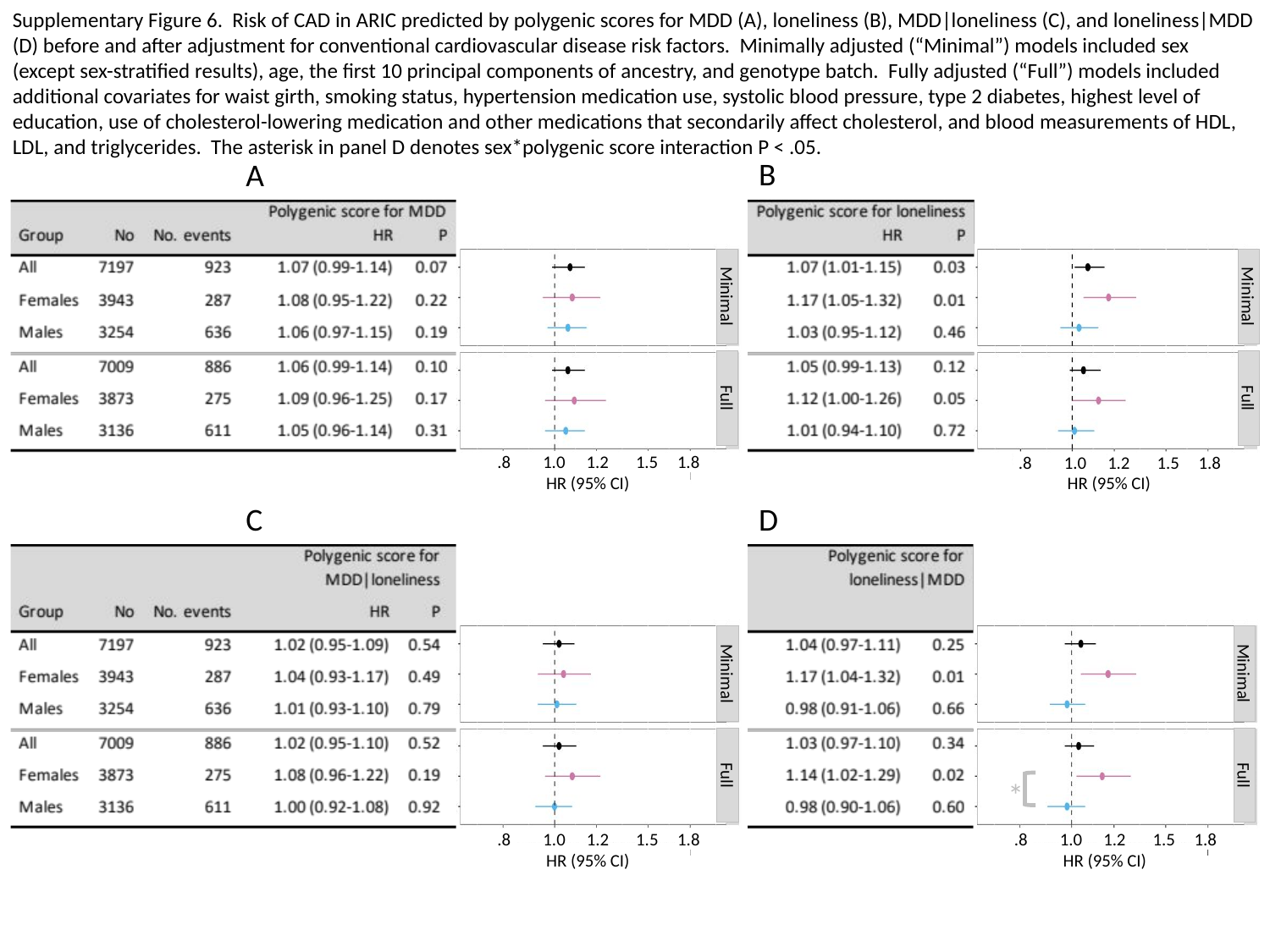

Supplementary Figure 6. Risk of CAD in ARIC predicted by polygenic scores for MDD (A), loneliness (B), MDD|loneliness (C), and loneliness|MDD (D) before and after adjustment for conventional cardiovascular disease risk factors. Minimally adjusted (“Minimal”) models included sex (except sex-stratified results), age, the first 10 principal components of ancestry, and genotype batch. Fully adjusted (“Full”) models included additional covariates for waist girth, smoking status, hypertension medication use, systolic blood pressure, type 2 diabetes, highest level of education, use of cholesterol-lowering medication and other medications that secondarily affect cholesterol, and blood measurements of HDL, LDL, and triglycerides. The asterisk in panel D denotes sex*polygenic score interaction P < .05.
B
A
Minimal
Full
.8
1.0
1.2
1.5
1.8
HR (95% CI)
Minimal
Full
.8
1.0
1.2
1.5
1.8
HR (95% CI)
C
D
Minimal
Minimal
Full
.8
1.0
1.2
1.5
1.8
HR (95% CI)
Full
*
.8
1.0
1.2
1.5
1.8
HR (95% CI)
